# S100A10 Overexpression Correlates with Adverse Prognosis, Tumor Microenvironment, and Aggressive Behavior *In Vitro* and *In Vivo* of Cervical Cancer

**DOI:** 10.7150/jca.87689

**Published:** 2023-09-11

**Authors:** Yue-Chen Zhao, Tie-Jun Wang, Li-Zhen She, Jie Cui, Chao-He Zhang

**Affiliations:** 1 Department of Radiation Oncology, The Second Hospital of Jilin University, Changchun, Jilin 130041, P.R. China.; 2 Department of Hematology and Oncology, The Second Hospital of Jilin University, Changchun, Jilin 130041, P.R. China.

**Keywords:** S100 Calcium Binding Protein A10 (S100A10), Cervical carcinoma, Cancer Biomarker, Prognosis, Tumor immune microenvironment

## Abstract

**Background:** The incidence of cervical squamous cell carcinoma and endocervical adenocarcinoma (CESC) is increasing in women. S100A10 overexpression is commonly reported in various malignancies and is closely associated with tumor cell characteristics and prognosis.

**Methods:** The expression of S100A10 and its prognostic relevance were assessed utilizing RNA-seq data from The Cancer Genome Atlas. S100A10 regulation of CESC cell growth and migration was investigated using CCK-8, colony-forming, and Transwell-based approaches. Xenograft model mice were used to examine the *in vivo* effects of S100A10, and differentially expressed genes (DEGs) linked to S100A10 were identified to explore its functional role in oncogenesis. Associations between S100A10 levels, chemosensitivity, and the immune microenvironment were assessed, and the mutational and methylation status of S100A10 was evaluated using the cBioPortal and MethSurv databases, respectively.

**Results:** S100A10 was upregulated in CESC samples, and higher S100A10 mRNA levels were associated in poor prognostic outcomes. The area under the curve for S100A10 when diagnosing CESC was 0.935, and S100A10 was found to regulate tumor cell proliferation and metastasis both *in vitro* and *in vivo*. Overall, 1125 DEGs enriched in crucial CESC progression-associated signaling pathways were identified. S100A10 expression was also associated with the intratumoral immune microenvironment and immune checkpoint activity. Patients expressing elevated S100A10 levels exhibited distinct chemotherapeutic susceptibility, and methylation of the S100A10 gene was correlated with patient survival outcomes.

**Conclusion:** In summary, this research demonstrated that S100A10 plays a crucial role in regulating CESC development, prognosis, and the intratumoral immune microenvironment. Thus, S100A10 shows potential as a prognostic or diagnostic tool and as a potential target for CESC immunotherapy.

## Introduction

Cervical cancer (CC) remains the second most prevalent form of gynecological malignancy and the fourth most common global cause of cancer-related death among women 1. The human papillomavirus vaccine has helped reduce CC incidence in many developed nations, but CC incidence rates nonetheless remain high in less developed areas, particularly among younger women. Indeed, CC occurs at rates 7- to 9-fold higher in developing nations as compared to developed nations, with a mortality rate that is 18-fold higher 2. There is thus a pressing need to better define biomarkers linked to CC patient prognosis in an effort to better support the diagnosis and treatment of this malignancy.

The S100 protein family member S100 Calcium Binding Protein A10 (S100A10) is comprised of two subunits that dimerize to form a functional unit 3. S100A10 can further undergo heterotrimerization with Annexin A2 (ANXA2) at the cell membrane where it functions as a regulator of cytoskeleton formation, endocytic activity, and the transport of cellular cargos 4. S100A10 can further function as a regulator of Toll-like receptor signaling activity, thereby regulating innate immune and inflammatory activity, in addition to controlling 5-hydroxytryptamine production (5-HT) through interactions with the receptor for 5-HT 5. Major physiological roles for S100A10 have been described in a series of physiological processes, and its dysregulation has been tied to diseases including schizophrenia, irritable bowel syndrome, and depression 6, 7.

S100A10 has also recently been tied to the incidence and progression of various forms of cancer. Notably, the combination of S100A10 and ANXA2 exhibits plasminogen regulatory activity, promoting the activity of urokinase- and tissue-type plasminogen and the consequent conversion of plasminogen into plasmin. This, in turn, can cause matrix metalloproteinase activation, extracellular matrix degradation, and the consequent growth and invasivity of tumors 8. Aberrant S100A10 upregulation has been reported in lung, gastric, ovarian, and breast cancers wherein it is associated with poorer prognostic outcomes 9-12. To date, there have been no detailed analyses of S100A10 expression in cervical squamous cell carcinoma and endocervical adenocarcinoma (CESC), and further work is vital to clarify its biological activity in this deadly oncogenic context.

To better understand the prognostic and diagnostic significance of S100A10 expression and its functional relevance in CESC, data from The Cancer Genome Atlas (TCGA) were herein analyzed and supported through *in vitro* and *in vivo* tests of the role of S100A10 in cancer. Additionally, we investigated the associations between S100A10 expression and various features, including chemotherapeutic susceptibility, immune checkpoint expression, and the immune microenvironment. Our findings lay a firm foundation for future studies aiming to clarify the role of S100A10 in CESC and determine its potential as a diagnostic or therapeutic target for this type of cancer.

## Materials and Methods

### TCGA analyses

The TCGA platform was queried to download data pertaining to patients with CESC including S100A10 mRNA levels as well as important clinicopathological details such as age, TNM staging, clinical staging, and other factors. The CESC patients were divided into high S100A10- and low S100A10-expression groups according to the median level of S100A10 mRNA expression.

### Clinical sample collection

Pairs of CESC patient tumors and healthy adjacent tissues were collected between January and August of 2022 from patients undergoing surgery at The Second Hospital of Jilin University. The patients were diagnosed with CESC at the first diagnosis, and all included patients exhibited a clear pathological diagnosis without having undergone any treatment before surgery. The study was conducted in accordance with the Declaration of Helsinki, and the study protocol was approved by the Medical Ethics Committee (Second Hospital of Jilin University). Informed consent was obtained from all subjects for the tissue specimen acquisition.

### Immunohistochemical (IHC) analysis

After having been cut to yield 4 μm sections, tissue sections were deparaffinized with xylene (Beijing Chemical Plant, China), rehydrated with an ethanol gradient (Beijing Chemical Plant), and treated with 3% H_2_O_2_ (Beyotime, China) prior to antigen retrieval at room temperature (RT). Sections were blocked for 30 min at RT using 5% BSA (Abcam, UK), incubated overnight with anti-S100A10 (Abcam) at 4°C, probed for 30 min using a secondary antibody (Abcam), and color was developed using DAB (Abcam) prior to hematoxylin counterstaining (Abcam), ethanol gradient dehydration, neutral resin fixation, and imaging *via* light microscopy.

### Western immunoblotting

The tissues were cut into small pieces and homogenized in RIPA lysis buffer (Solarbio, China) to extract the protein. Subsequently, the protein concentration was assessed using the Bicinchoninic acid method. The extracted proteins (20 μg/sample) were then separated on 10% SDS-PAGE gels (Beyotime) and transferred to PVDF membranes (Millipore, USA). After blocking with 5% skimmed milk for 1 h at RT, the membranes were incubated with the primary antibody against S100A10 (1:1000, Abcam) or the internal protein standard β-actin (1:1000, Abcam) overnight at 4°C. The following day, the membranes were incubated with the secondary antibody (1:10000, Abcam) for 2 h at RT and were visualized using the BeyoECL Plus Kit (Beyotime).

### Cell culture and transfection

The human HeLa cell line (Procell, China) was cultured in MEM medium (Procell) supplemented with 10% fetal bovine serum (FBS) (Gemini, USA) in a humidified incubator at 37°C with 5% CO_2_. To establish stably transfected cell lines with downregulated S100A10 expression, HeLa cells were infected with lentiviral vectors containing either S100A10 short-hairpin RNA (sh-S100A10) or a corresponding negative control (sh-NC), which were synthesized by Genechem (China). The transfection procedure was conducted in HeLa cells using the Polybrene (Hanbio, China). After the stably transfected cells were selected using puromycin according to the manufacturer's protocol, they were collected and utilized for subsequent experiments. The transfection efficiency was assessed through RT-qPCR analysis.

### Quantitative Real-time PCR (RT-qPCR) assays

Total RNA was isolated using the Triquick Reagent (Solarbio), followed by reverse transcription of S100A10 using the SweScript RT I First Strand cDNA Synthesis Kit (Servicebio, China). The synthesized cDNA was mixed with 2×SYBR Green qPCR Master Mix (Low ROX) (Servicebio) and specific primers for RT-qPCR analysis, followed by amplification using at ABI PRISM 7000 Sequence Detection System (Applied Biosystems, USA). The 2^-ΔΔCT^ method was used to calculate the relative expression of S100A10, with GAPDH as an internal control. The primer sequences used for S100A10 were: 5'- GGCTACTTAACAAAGGAGGACC' (forward) and 5'- GAGGCCCGCAATTAGGGAAA-3' (reverse).

### Cell Counting Kit-8 (CCK-8) assays

CCK-8 assays were used to measure cell viability as an assessment of the proliferative ability of the cells. Following transfection, HeLa cells were seeded in 96-well plates at a density of 1×10^5^ cells/mL and incubated at 37°C for 48 h. A CCK-8 assay kit (Meilunbio, China) was used to perform the assays, according to the provided instructions, with the addition of 10 μL of the CCK-8 reagent to each well. This procedure was repeated five times using different wells. After continuous incubation for 3 h at 37°C, a microplate reader (Bio-Rad, model 550, USA) was used to measure the absorbance at a wavelength of 450 nm. The resulting absorbance values were used to calculate cell viability, which was normalized to the values of the control group (100%).

### Colony forming assays

Cell proliferation was assessed using colony forming assays. Briefly, transfected cells were seeded in 6-well plates at a density of 500 cells per well. After 10 days of cell culture, the medium was replaced, and the cells were washed with PBS. The colonies were then fixed with 4% paraformaldehyde (Beijing Chemical Plant) for 30 min and stained with 2% crystal violet (Beyotime) for 20 min. Images of the colonies were captured, and visible colonies (≥ 50 cells) were observed and counted using an optical microscope (Nikon Eclipse Ti2, Japan).

### Wound healing assay

The migratory capacity of HeLa cells was assessed using wound healing assays. Transfected HeLa cells were seeded in 6-well plates and allowed to grow for 48 h until almost confluent. Sterilized 20 μL pipette tips were used to create longitudinal scratches on the cell monolayers and non-adherent cells were removed by washing with PBS. At 0 h and 24 h after the start of the experiment, the width of the scratches was imaged using an optical microscope at 40× magnification, and the distance migrated by the cells was measured using ImageJ software. The formula used for calculation was: cell mobility = (wound width at 0 h - wound width at 24 h)/wound width at 0 h.

### Transwell assays

For the determination of the invasive capacity of HeLa cells, Transwell chambers (Corning, USA) were pre-coated with Matrigel (Solarbio) according to the manufacturer's instructions. In each experimental group, transfected HeLa cells in 200 μL of serum-free medium were seeded in the upper compartment of the chamber, while the lower compartment was filled with 600 μL of MEM medium containing 10% FBS. After 24 h, the cells that had invaded and migrated to the lower chamber were fixed with 4% paraformaldehyde (Beyotime) and stained with Giemsa Stain solution (Solarbio). The invasive cells in five randomly selected regions were visualized and counted using an inverted microscope at 100× magnification.

### *In vivo* xenograft model

Animal studies were conducted with the approval of Animal Ethics Committee (College of Basic Medicine of Jilin University). Female BALB/cA-nu nude mice (Changsheng Biotechnology, China), aged 6-8 weeks, were bred in a specific pathogen-free environment. Stably transfected HeLa cells in the logarithmic growth phase were digested with trypsin and then suspended in PBS at a concentration of 1 × 10^8^ cells/ml. One hundred microliters of the cell suspension were injected subcutaneously on the dorsal side of the hind limbs of the nude mice using a syringe. Transfected sh-S100A10 and sh-NC HeLa cells were injected into three mice, respectively. Approximately 7 days post-inoculation, palpable nodules of detectable size were observed beneath the skin, with the nodules progressively increasing in size over time. The tumor dimensions, including the longest diameter (L) and the shortest diameter (W), were periodically measured using calipers. On the 27th day, the mice were humanely euthanized by cervical dislocation, and the transplanted tumors were excised, photographed, weighed, and measured. Tumor volumes were determined using the formula (L × W^2^)/2. The harvested tumor tissues were then divided into two parts, one of which was frozen in liquid nitrogen, while the other was fixed in a 4% tissue fixation solution for a minimum of 24 h.

### Differentially expressed gene (DEG) identification and analysis

DEGs related to S100A10 in CESC were identified utilizing the LinkedOmics database and CESC patient data from the TCGA database. The selection criteria for DEGs included a fold-change > 1.5 and an FDR-corrected P-value < 0.05. Volcano plots were generated for visual representation of the DEG identification results. Subsequently, a heatmap was constructed, incorporating the 50 most significantly up- and downregulated genes, to provide a comprehensive overview of the expression patterns of the DEGs. To examine the biological implications of the DEGs, functional enrichment analyses using Gene Ontology (GO) and Kyoto Encyclopedia of Genes and Genomes (KEGG) were performed to provide a deeper understanding of their associated biological functions and pathways.

### Immune infiltration assessment

The R GSVA package was used for a single-sample GSEA analysis of immune cell infiltration to evaluate infiltration by 24 immune cell types and the expression of immune checkpoint molecules such as LAG3, CTLA4, TIGIT, SIGLEC15, HAVCR2, CD274, PDCD1, and PDCD1LG2. Differences in the expression of these molecules were compared between individuals expressing low and high levels of S100A10.

### Chemotherapeutic susceptibility analyses

The Cancer Drug Sensitivity Genome Database (https://www.cancerrxgene.org/) was used with the R "pRRophetic" package to predict chemotherapeutic susceptibility in CESC patients. The half-maximal inhibitory concentration (IC_50_) values of individual samples were calculated using the ridge regression method, and all analytical parameters were set to standard values.

### Epigenetic analyses

Changes in the S100A10 gene in the TCGA (Firehose Legacy and PanCancer Atlas) database were evaluated using cbioportal (www.cbioportal.org). Differences in prognostic scores were compared using Kaplan-Meier plots and log-rank tests. The association between the methylation of individual CpG S100A10 residues and survival was assessed by searching methylation-related data using the MethSurv database (bit.cs.utt.ee/MethSurv/).

### Statistical analysis

Statistical evaluations were performed using R version 4.2.3. Group comparisons were performed using Wilcoxon tests, while pROC software was used to construct receiver operating characteristic (ROC) curves to determine the significance of S100A10 expression levels. The relationship between S100A10 levels and clinical characteristics in patients with CESC was assessed using Chi-square tests. Additionally, Kaplan-Meier curves were constructed to compare differences in the overall survival (OS) between groups, and P-values were calculated using the log-rank test of the R-Survival package. Error bars in the bar and box plots represent standard deviation (SD). The significance threshold was set at P < 0.05.

## Results

### Evaluation of the expression patterns of S100A10 in malignancy

**Figure 1:**
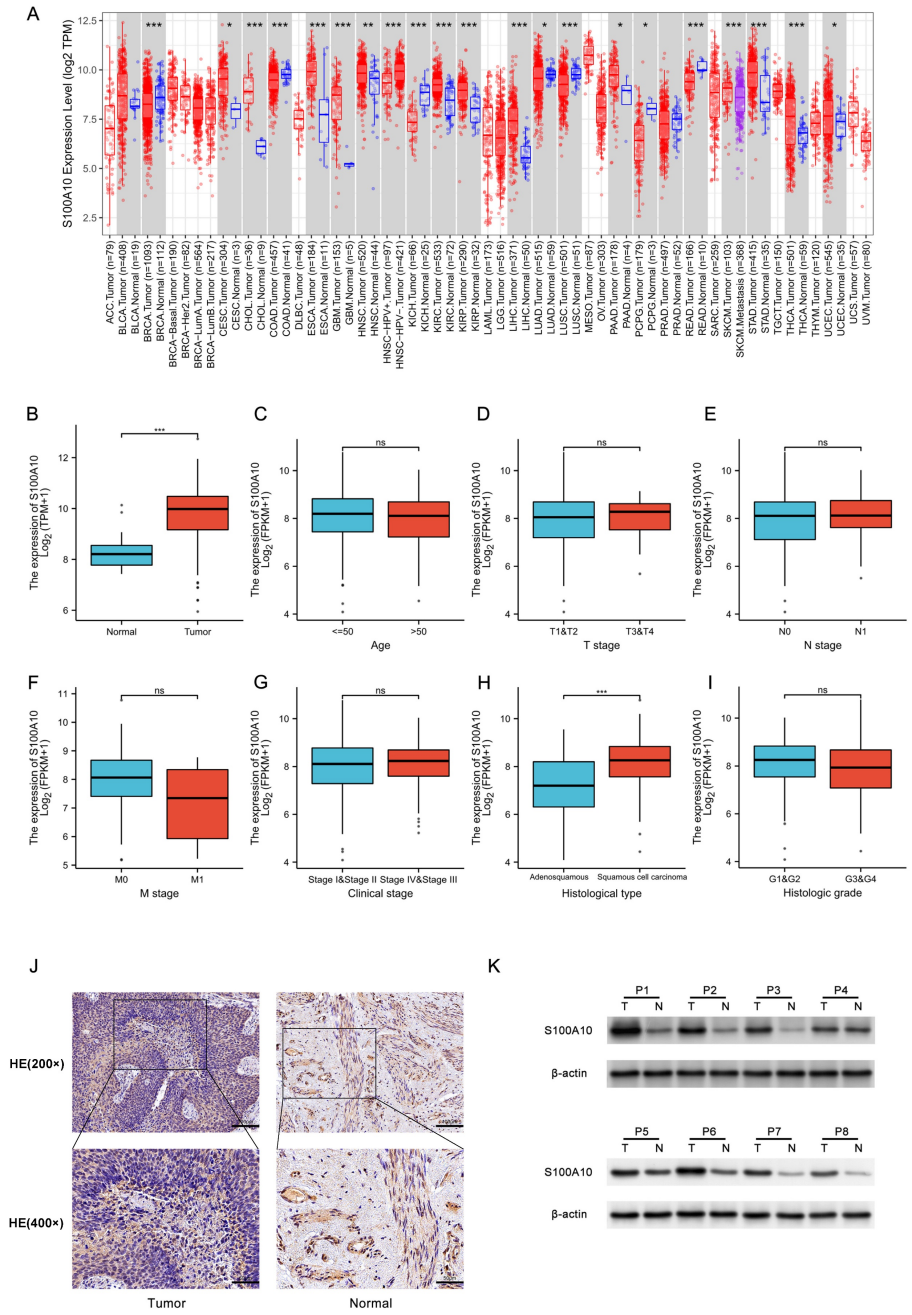
S100A10 mRNA expression in various contexts. (a) Pan-cancer evaluation using the TIMER 2.0 database, including both tumor and non-tumor samples. (b) Comparison of S100A10 mRNA expression levels between CESC tissues and healthy controls. (c-i) Associations between S100A10 mRNA expression and clinicopathological parameters of CESC patients, including age, histologic grade, TNM classification, clinical staging, and histologic type. Assessment of S100A10 expression *via* (j) IHC staining (×200 and ×400) and (k) Western immunoblotting in paired CESC tumor and paracancerous tissues. * P < 0.05, ** P < 0.01, *** P < 0.001; ns = not significant.

Initially, the expression of S100A10 was assessed across all cancers in the TIMER 2.0 database, revealing it to be overexpressed in CESC, CHOL, ESCA, GBM, HNSC, KIRC, KIRP, LIHC, PAAD, STAD, THCA, and UCEC, whereas S100A10 downregulation was detected in BRCA, COAD, KICH, LUAD, LUSC, PCPG, and READ (Fig. 1a). Higher S100A10 expression was evident in CESC patient tumors relative to healthy tissues (P < 0.001) (Fig. 1b), and correlations were detected between the expression of this gene and clinical parameters including histological tumor type (P < 0.001) (Fig. 1c-i). In contrast, S100A10 levels were unrelated to patient age, or to the tumor T, N, M, G, or clinical stages (P > 0.05). The upregulation of S100A10 in tumors was further confirmed through both IHC and Western immunoblotting, which were consistent with the findings from the TCGA database (Fig. 1j-k).

### Analyzing the relationship between S100A10 expression and clinical data of CESC patients

According to the expression level of S100A10, CESC patients in the TCGA database were divided into two groups, and the clinical characteristics such as age, TNM classification, G classification, and clinical classification of patients in each subgroup were compared (Table 1). Analysis of the results showed that the S100A10 mRNA levels were significantly correlated with histological type (P < 0.001), OS event (P = 0.043), and primary therapy outcome (P = 0.019).

### The diagnostic efficacy of S100A10 in CESC

**Figure 2:**
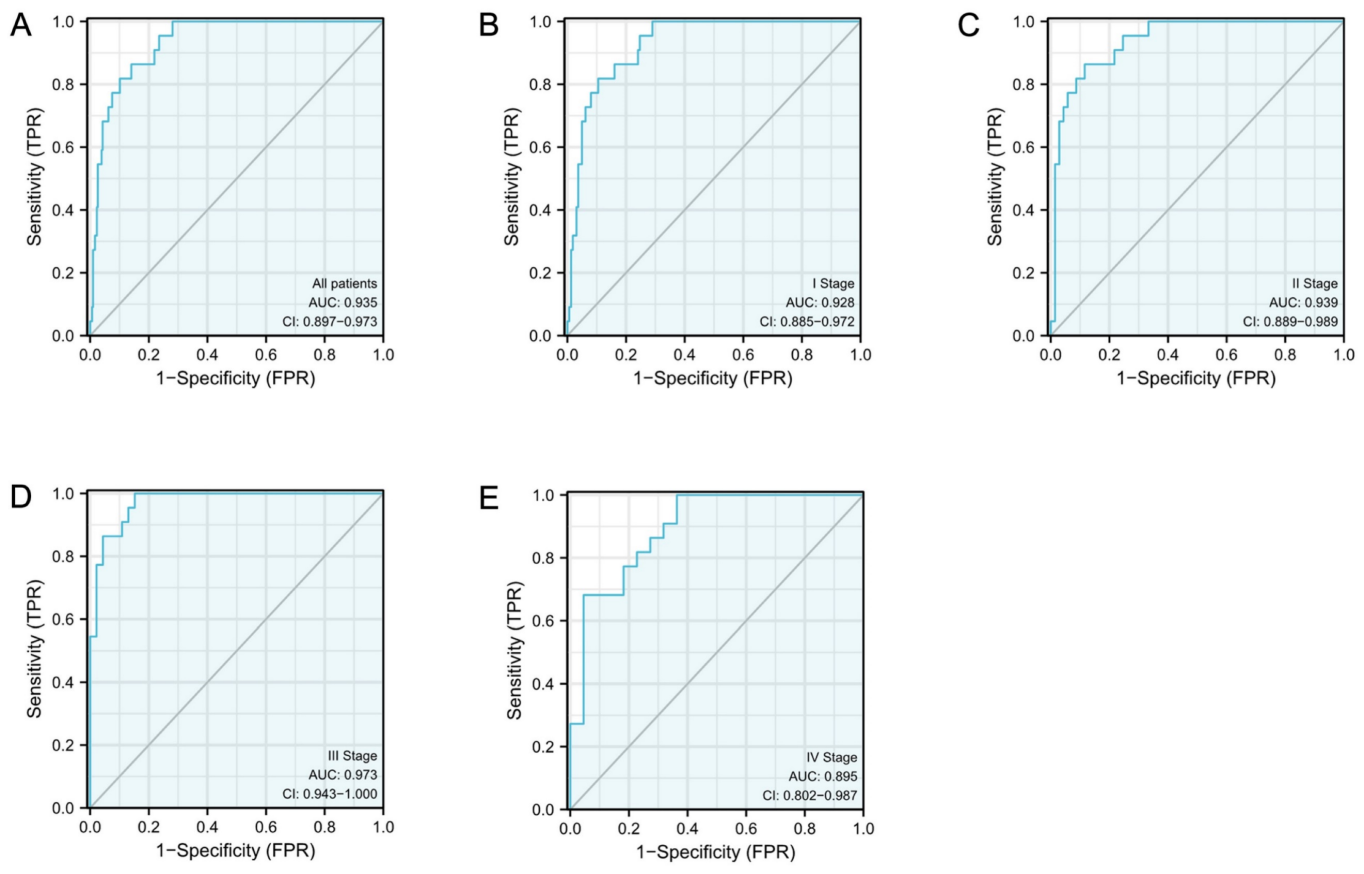
The S100A10 ROC curve analyses in CESC patients. Normal samples are compared to (a) tumors, (b) stage I tumors, (c) stage II tumors, (d) stage III tumors, and (e) stage IV tumors.

ROC curves were subsequently constructed based upon S100A10 expression in CESC (Fig. 2a), revealing a calculated area under the curve (AUC) value of 0.935 consistent with superior diagnostic utility. Similarly, the AUC values for specific CESC tumor stages were generated, with respective values of 0.928, 0.939, 0.973, and 0.895 for stage I, II, III, and IV disease (Fig. 2b-e). ROC curves were subsequently generated based upon the S100A10 expression in CESC. This showed an AUC value of 0.935, confirming its exceptional diagnostic efficacy (Fig. 2a). Moreover, specific AUC values were computed for different stages of CESC, yielding values of 0.928, 0.939, 0.973, and 0.895 for stage I, II, III, and IV disease, respectively (Fig. 2b-e).

### Prognostic role of S100A10 in CESC

**Figure 3:**
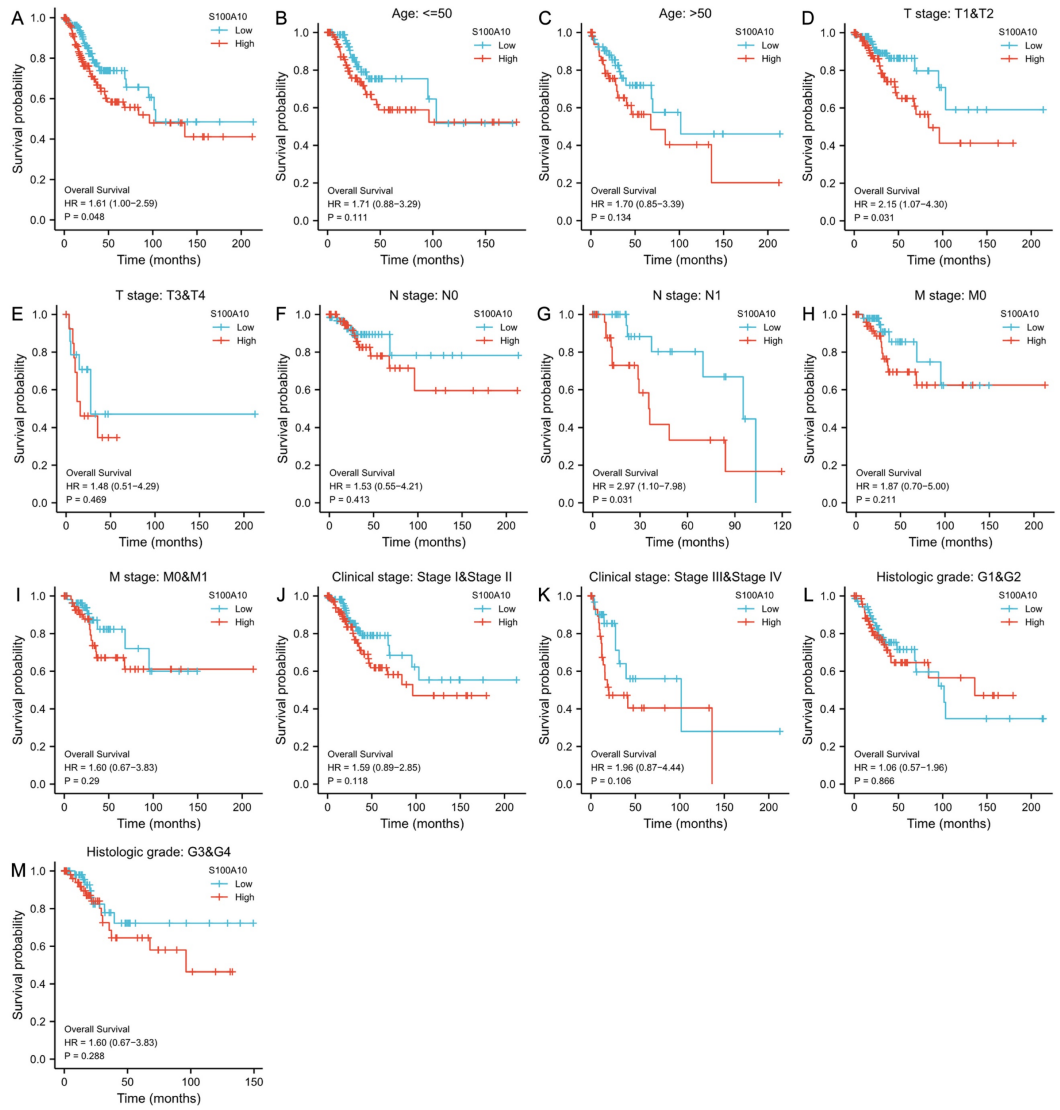
Association of S100A10 expression with CESC patient survival. (a) Kaplan-Meier analyses of OS outcomes in CESC patients from the TCGA database. (b-m) Survival subgroup analyses based on CESC patient clinical characteristics, including age, histologic grade, TNM staging, and clinical staging.

Using Kaplan-Meier curves, the prognostic relevance of S100A10 mRNA levels in CESC was further explored, highlighting a link between the overexpression of S100A10 and poorer patient OS (P = 0.048) (Fig. 3a). This was also true in subgroup analyses, which revealed an association between S100A10 levels and OS in individuals with T1/T2 stage (P = 0.031), and N1 stage disease (P = 0.031). In contrast, S100A10 levels were unrelated to survival outcomes in other subgroups (P > 0.05) (Fig. 3b-m). Univariate analyses indicated that poorer OS was significantly associated with T (P < 0.001), N (P = 0.002), M (P = 0.023), and clinical stage (P < 0.001) and with the expression of S100A10 (P = 0.029), while multivariate tests confirmed that T stage was independently associated with CESC patient prognosis (hazard ratio = 5.276, P = 0.024) (Table 2).

### Experimental confirmation of the functional significance of S100A10 in CESC

**Figure 4:**
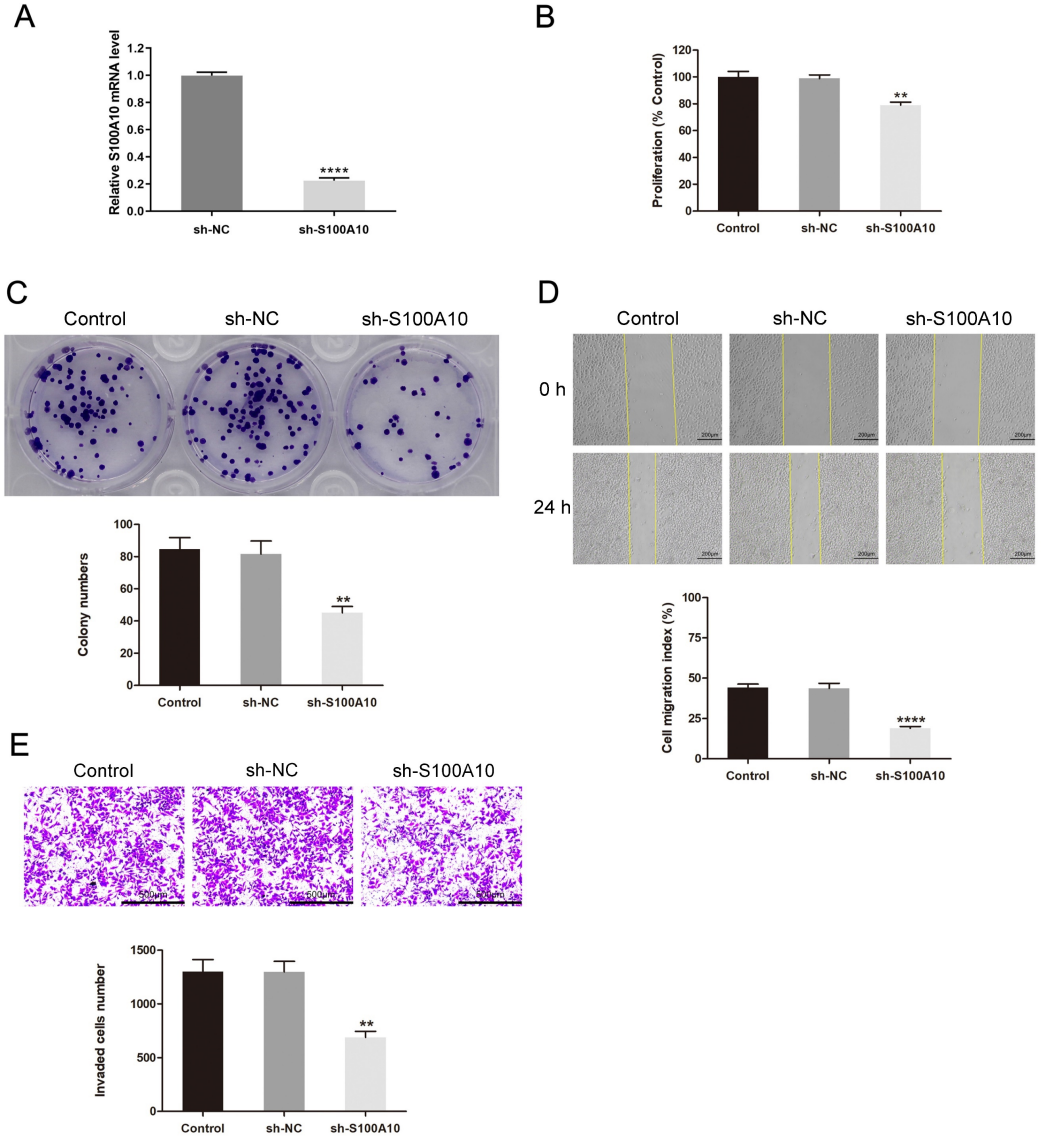
Effects of S100A10 knockdown on CESC cell growth and metastatic potential. (a) The transfection efficiency of sh-S100A10 determined by RT-qPCR. HeLa cells transfected using sh-NC or sh-S100A10 constructs were analyzed in (b) CCK-8, (c) colony forming, (d) wound healing, and (e) Transwell invasion assays. ** P < 0.01, **** P < 0.0001.

**Figure 5:**
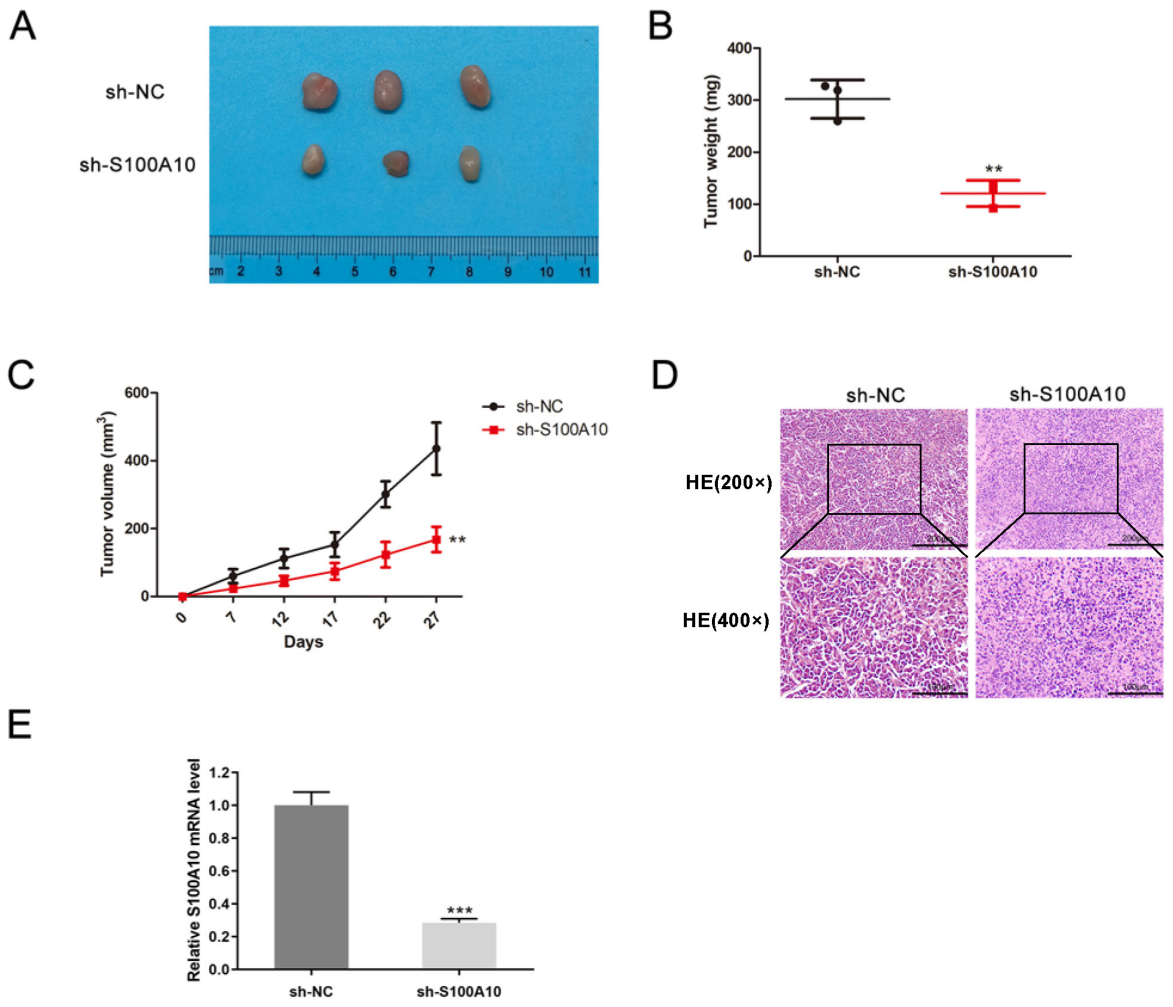
Suppression of tumor growth in CESC models by S100A10 knockdown. (a) Tumor size, (b) weight, (c) volume, (d) histological grading (×200 and ×400), and (e) S100A10 mRNA expression were detected. Results are means ± SD. ** P < 0.01, *** P < 0.001.

Next, S100A10 silencing was performed in HeLa cells by shRNA, the efficiency of silencing was confirmed by RT-qPCR (Fig. 4a). The CCK-8 and colony forming assays indicated that this knockdown resulted in impaired cellular proliferation as compared to control construct transfection (P < 0.01) (Fig. 4b-c). Loss of S100A10 expression also impaired the migratory (P < 0.0001) (Fig. 4d) and invasive activity of these cells (P < 0.01) (Fig. 4e). Consistently, *in vivo* xenograft experiments revealed that on day 27, the tumor weights in the sh-NC and sh-S100A10 groups were 302.07 ± 36.91 mg and 120.93 ± 25.00 mg (P < 0.01) (Fig. 5a-b), respectively, whereas the respective tumor volumes were 435.55 ± 76.63 mm^3^ and 167.81 ± 37.28 mm^3^ (P < 0.01) (Fig. 5c). Following S100A10 knockdown, tumors exhibited a smaller nuclear proportion, less intense nuclear staining, and fewer mitotic images (Fig. 5d). Additionally, RT-qPCR results revealed notably reduced expression levels of S100A10 in S100A10-knockdown tumors (P < 0.001) (Fig. 5e).

### Functional landscape of S100A10-associated DEGs

**Figure 6:**
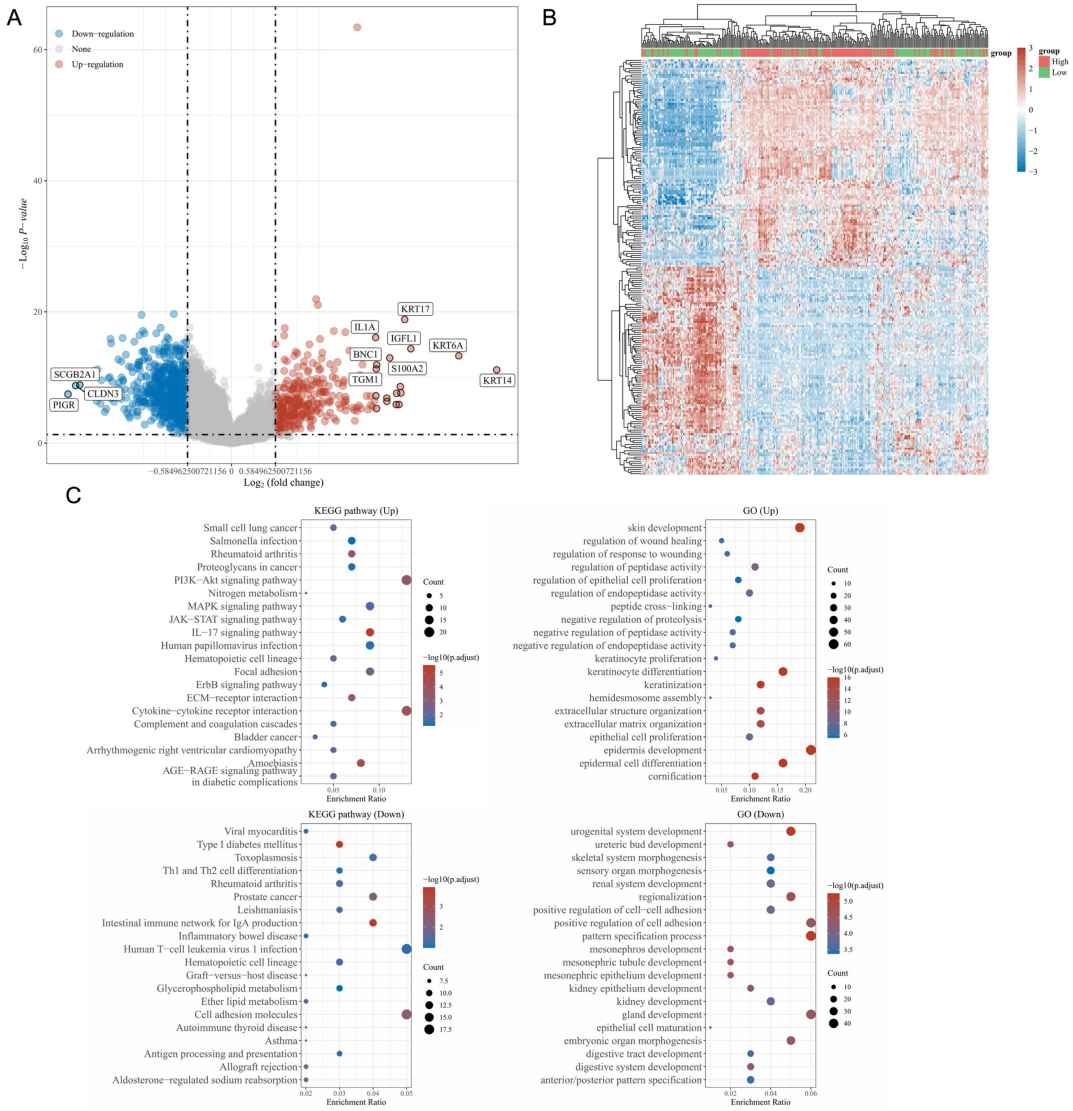
Enrichment analyses of S100A10-associated DEGs. (a) Volcano plot showing the identified DEGs, with upregulated genes colored red and downregulated genes colored blue. (b) Heatmap of DEGs, with upregulated genes in red and downregulated genes in blue. (c) KEGG and GO enrichment analyses of DEGs.

Using the LinkedOmics database, genes specifically expressed in a manner associated with S100A10 expression were identified including 315 and 810 DEGs that were respectively positively and negatively associated with S100A10 expression (Fig. 6a). Of these genes, the 50 most positively and negatively correlated were used to construct a heatmap (Fig. 6b). KEGG and GO enrichment analyses of these genes suggested that they were involved in the PI3K-Akt, IL-17, human papilloma virus (HPV) infection, extracellular matrix (ECM)-receptor interaction, cytokine-cytokine receptor interaction, and cell adhesion KEGG pathways in addition to being associated with GO terms including epidermis and keratinocyte growth and specialization, extracellular matrix organization, and negative regulation of proteolysis and positive regulation of cell adhesion (Fig. 6c).

### Correlation between S100A10 levels and the intratumoral immune microenvironment

**Figure 7:**
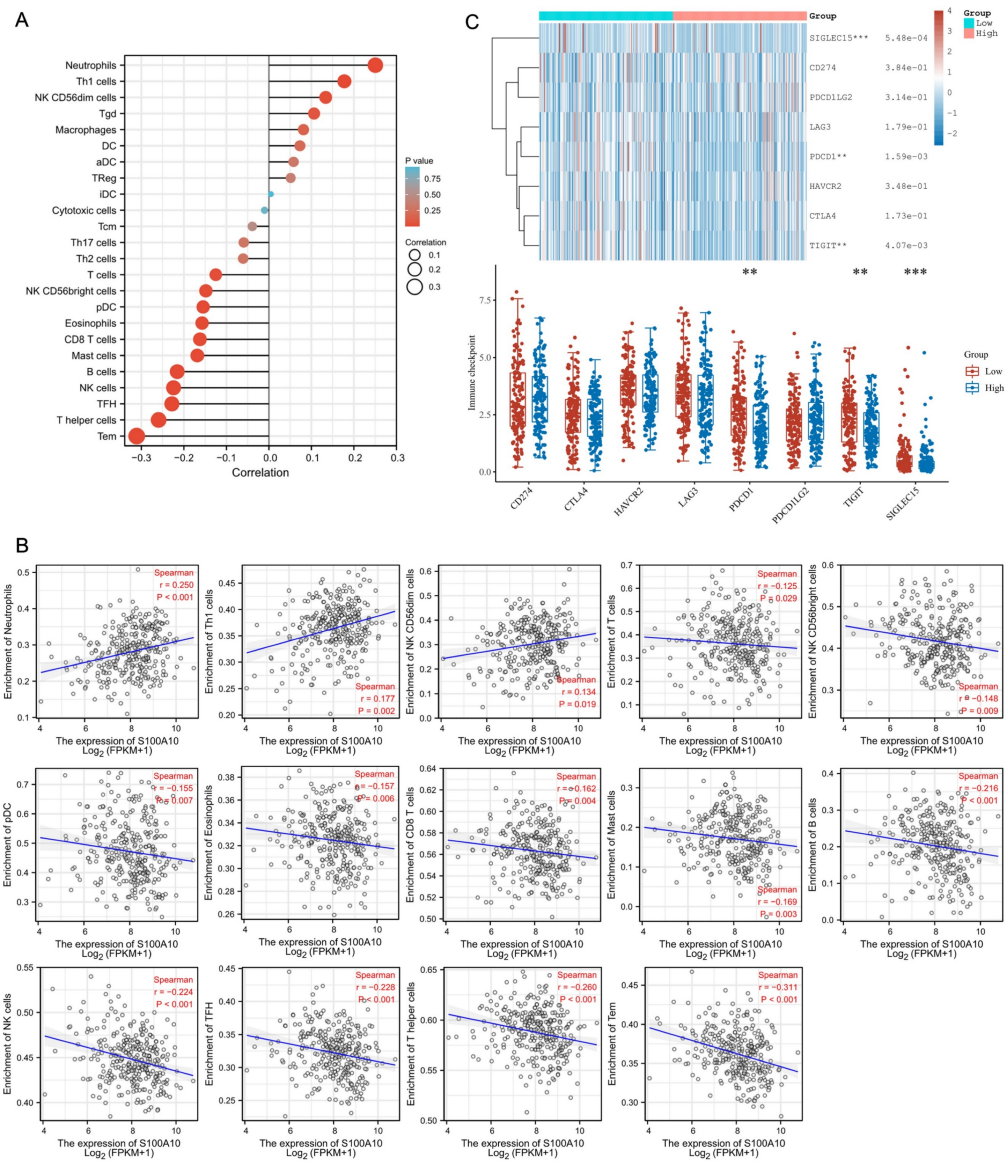
Tumor microenvironment immune cell infiltration analyses. (a) A forest plot. (b) Spearman correlation analyses. (c) Assessment of immune checkpoint marker expression in patient groups with low and high S100A10 levels. ** P < 0.01, *** P < 0.001.

Analyses of the relationship between S100A10 transcription and intratumoral immune microenvironment in CESC patients were next performed. A positive correlation was detected between S100A10 levels and infiltration of these tumors by Neutrophils (P < 0.001), Th1 cells (P = 0.002), and NK CD56dim cells (P = 0.019), while S100A10 levels were negatively correlated with infiltration by T cells (P = 0.029), NK CD56bright cells (P = 0.009), pDC (P = 0.007), Eosinophils (P = 0.006), CD8 T cells (P = 0.004), Mast cells (P = 0.003), B cells (P < 0.001), NK cells (P < 0.001), TFH cells (P < 0.001), T helper cells (P < 0.001), and Tem cells (P < 0.001) (Fig. 7a-b). To investigate the predictive utility of S100A10 with respect to immunotherapy responses, correlation analysis between S100A10 expression and immune checkpoints was performed. These data revealed that S100A10 expression was negatively correlated with the expression of PDCD1, TIGIT and SIGLEC15 (Fig. 7c).

### S100A10 expression is linked with CESC patient chemotherapeutic susceptibility

**Figure 8:**
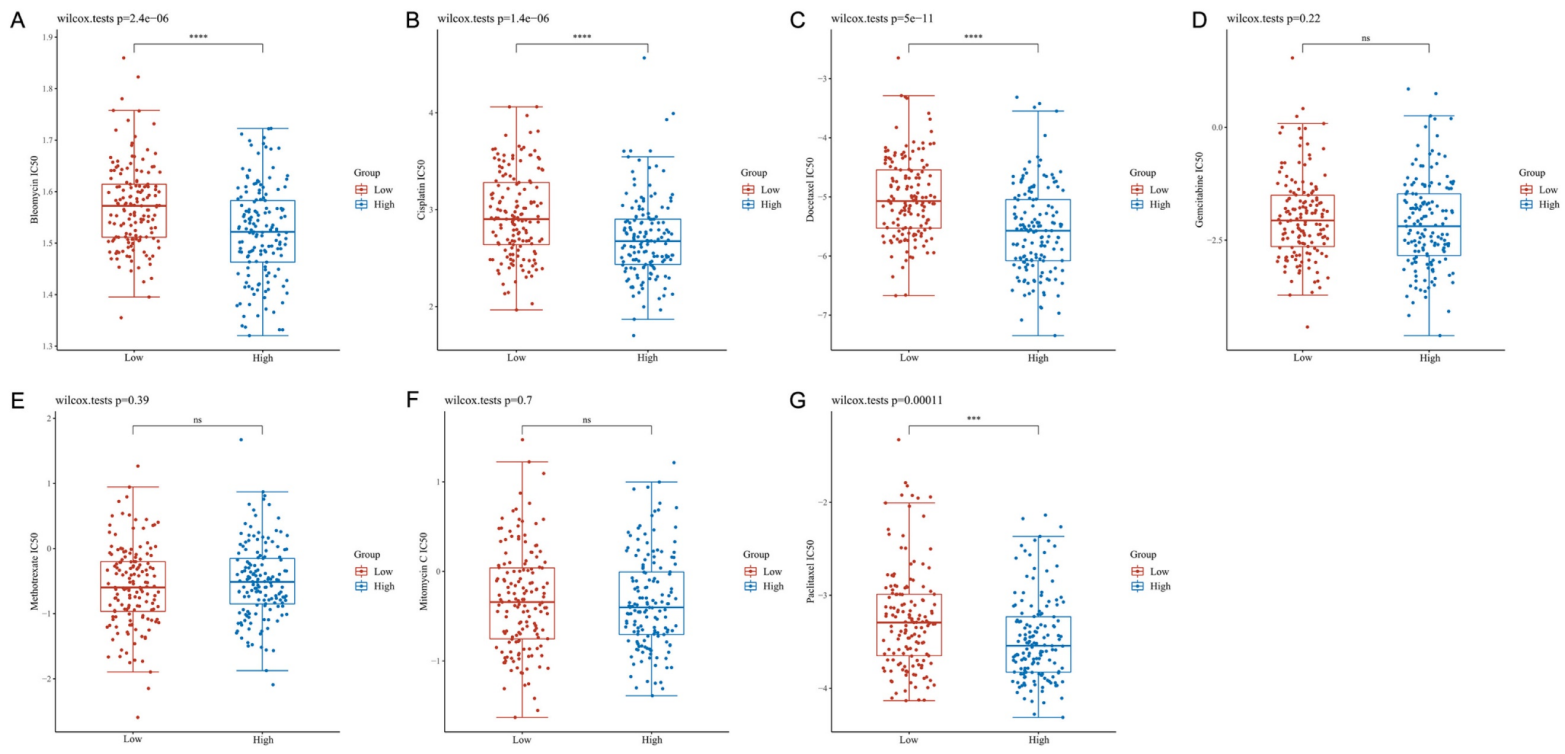
Chemotherapeutic susceptibility analyses. IC_50_ values for antineoplastic drugs: (a) Bleomycin, (b) Cisplatin, (c) Docetaxel, (d) Gemcitabine, (e) Methotrexate, (f) Mitomycin C, and (g) Paclitaxel. ns = not significant.

The IC_50_ values for clinically available chemotherapeutic drugs for CESC were compared in different patient groups to assess chemotherapeutic susceptibility. This approach revealed that patients expressing high S100A10 levels exhibited reduced IC_50_ values for Bleomycin (P = 2.4e-06), Cisplatin (P = 1.4e-06), Docetaxel (P = 5e-11) and Paclitaxel (P = 0.00011), whereas these values were not significantly altered for Gemcitabine (P = 0.22), Methotrexate (P = 0.39), or Mitomycin C (P = 0.70) (Fig. 8a-g).

### Altered S100A10 gene methylation in CESC

**Figure 9:**
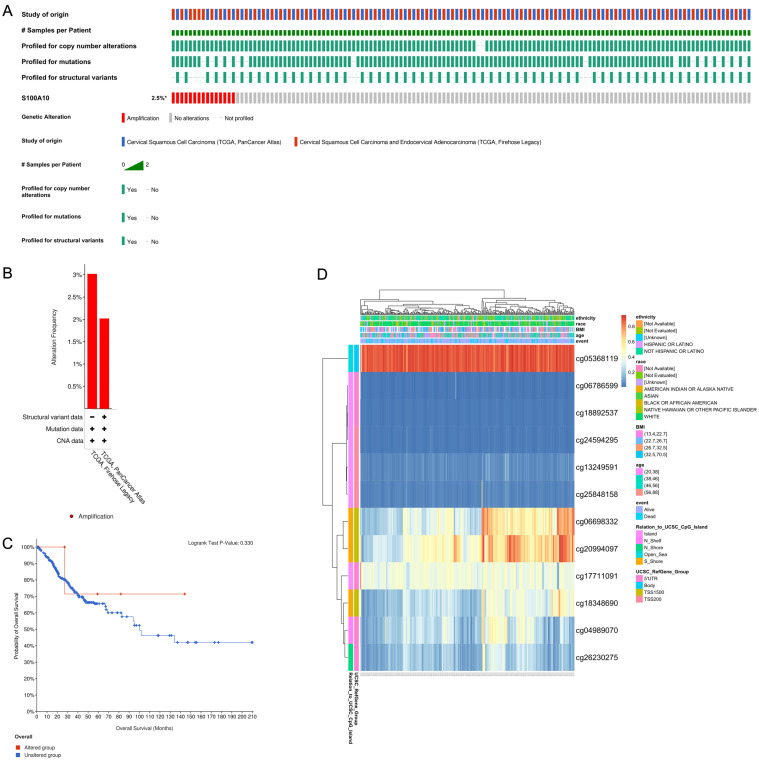
CESC patient changes in S100A10 genetic sequences and methylation status. (a) Visual summary of S100A10 alterations using OncoPrint. (b) A summary of CESC-related S100A10 variations from the TCGA, Firehose Legacy, and TCGA, PanCancer Atlas databases. (c) Kaplan-Meier plots comparing OS between patients with and without alterations in the S100A10 gene sequence. (d) Assessment of S100A10 methylation and expression levels.

Analysis of alterations of the S100A10 gene was then conducted in CESC patient samples using two databases (Fig. 9b). It was found that while S100A10 genetic alterations were apparent in 2.5% of cases (Fig. 9a), these alterations were not significantly associated with patient survival outcomes (P = 0.330) (Fig. 9c). Furthermore, the prognostic relevance of specific CpG residues in S100A10 methylation was explored using the MethSurv tool. Among the identified CpG residues, cg05368119 exhibited the highest level of methylation (Fig. 9d). Importantly, five of these CpG residues were found to be associated with the prognosis of CESC patients. Notably, increased methylation levels of cg06786599 (P = 0.0072) and cg24594295 (P = 0.0363) were linked to poorer OS in CESC patients (Table 3).

## Discussion

CC represents a major threat to global public health, yet its impact disproportionately affects less developed nations. While CC-related mortality rates have been gradually declining in developed nations, over 85% of CC cases and 90% of associated deaths continue to occur in underprivileged regions of the globe 13. A range of factors may contribute to this disparity, such as pollution or poorer hygiene 14, 15. Overall, 60.3% and 57.38% of individuals diagnosed with CC are under the ages of 50 and 40, respectively 16. As this form of cancer continues to impact ever-younger individuals, the need for surgical intervention during the early stages of this disease as a means of improving patient outcomes and preserving fertility continues to grow increasingly important 17. It is thus critical that novel biomarkers be defined to aid in the precision diagnosis, treatment, and prognostic assessment of patients affected by CC.

The plasminogen receptor S100A10 plays a key role in plasminogen conversion into plasmin, thereby shaping extracellular proteolytic activity 18. S100A10 is closely tied to the regulator of diverse pathophysiological processes, and in oncogenic settings it can reportedly shape intratumoral angiogenesis, intratumoral circulation, and tumor cell invasivity, migration, and metastatic progression 19. Tumor-associated macrophages (TAMs) are increasingly known to act as key tumor microenvironment components that shape cancer-associated processes including angiogenic activity, the epithelial-mesenchymal transition, chemoresistance, immunosuppression, and the uptake and release of specific macromolecules 20. Swisher et al. determined that TAM surface S100A10 expression plays a key role in oncogenic progression 21. The Ras oncogene is also a major driver of many cancers, and S100A10 interactions with Ras can reportedly enhance tumor cell invasivity through basement membrane and extracellular matrix degradation 22. This prior evidence highlights the important roles that S100A10 can play in cancer, emphasizing its relevance as a target for therapeutic intervention efforts.

These findings emphasize the significance of S100A10 as a regulator of tumor growth, prognostic outcomes, and the composition of the tumor microenvironment. In this study, S100A10 overexpression was observed in CESC tumor samples in comparison with healthy tissues, and these findings were corroborated in a separate cohort of clinical samples. Notably, S100A10 levels showed potential as a biomarker to distinguish between different stages of CESC. High levels of S100A10 were also significantly associated with poorer OS in CESC patients. *In vitro* and xenograft experiments supported the pro-oncogenic role of S100A10, providing further evidence of its significance in this specific cancer context.

To better define the functional roles of S100A10 in the progression of CESC, key co-expressed genes were identified and subjected to functional enrichment analyses. These DEGs were enriched in KEGG pathways including the PI3K-Akt pathway, which relates to chemoresistance, angiogenesis, protein synthesis, and tumor inhibition 23. They were also related to ECM-receptor interactions and adhesion molecule activity associated with tumor invasivity and metastasis 24 and IL-17 signaling which can serve a pro-tumorigenic function through the activation of precancerous cells and the suppression of antitumor immunity 25. HPV infection directly contributes to CC incidence. GO analyses also indicated that these genes are associated with the regulation of cell adhesion, ECM organization, and proteolysis, in line with the above KEGG results. Accordingly, high S100A10 expression levels may shape CESC progression in part through these pathways.

A large body of recent research has focused on the composition of the tumor microenvironment and its association with immune activity, given the presence of many infiltrating T cells, B cells, macrophages, and NK cells within patient tumors 26. These cells can exert antitumor immune functionality and facilitate immune surveillance, but the dysregulation of appropriate immunological activity can enable tumors to evade appropriate immune-mediated destruction. Here, S100A10 expression was found to be negatively correlated with tumor infiltration by T cells, NK CD56bright cells, pDC, Eosinophils, CD8 T cells, Mast cells, B cells, NK cells, TFH cells, T helper cells, and Tem cells. Accordingly, S100A10 upregulation may be linked to immune cell infiltration in a manner conducive to tumor immune evasion.

Immune checkpoint molecules are present on immune cell surfaces and serve as essential regulators of immune cell activity, with monoclonal antibodies targeting these checkpoints having emerged as invaluable tools for treating many different advanced cancer types 27. Here, high levels of S100A10 expression were found to be related to worse checkpoint expression profiles, suggesting that this S100A10 upregulation may be predictive of poorer patient immunotherapeutic responses.

The unchecked growth of tumor cells due to oncogenic driver mutations is a hallmark of cancer. In this study, S100A10 mutations were detected in only 2.5% of all CESC patients. However, recent research has demonstrated that epigenetic alterations, specifically gene methylation, can provide valuable insights into cancer-related biomarkers. In many instances, DNA-based research has aided in the development of improved methods to diagnose, evaluate, or treat specific types of tumors 28, 29. Thus, this study identified key CpG residues that were methylated in a manner associated with CESC patient prognosis.

## Conclusion

In conclusion, this study identified S100A10 expression as a crucial biomarker in CESC patients, particularly relevant to the context of immunotherapeutic treatment. These findings provide a valuable foundation for further research directed towards defining the key pathways that determine the malignant behavior and progression of CESC tumors, ultimately assisting in the development of novel targeted therapies for this disease.

## Figures and Tables

**Table 1:** Correlative relationships between the expression of S100A10 and CESC patient clinical characteristics.

Characteristic	Variable	No. of patients	S100A10 mRNA expression		P value
			Low, n (%)	High, n (%)	
Age	<=50	188	91 (29.7%)	97 (31.7%)	0.557
	>50	118	62 (20.3%)	56 (18.3%)	
Weight	<=70	138	69 (24.9%)	69 (24.9%)	1.000
	>70	139	69 (24.9%)	70 (25.3%)	
T stage	T1	140	76 (31.3%)	64 (26.3%)	0.485
	T2	72	41 (16.9%)	31 (12.8%)	
	T3	21	8 (3.3%)	13 (5.3%)	
	T4	10	5 (2.1%)	5 (2.1%)	
N stage	N0	134	71 (36.4%)	63 (32.3%)	0.900
	N1	61	31 (15.9%)	30 (15.4%)	
M stage	M0	116	63 (49.6%)	53 (41.7%)	0.753
	M1	11	7 (5.5%)	4 (3.1%)	
Clinical stage	Stage I	162	87 (29.1%)	75 (25.1%)	0.621
	Stage II	69	33 (11%)	36 (12%)	
	Stage III	46	20 (6.7%)	26 (8.7%)	
	Stage IV	22	11 (3.7%)	11 (3.7%)	
Histological type	Adenosquamous	53	39 (12.7%)	14 (4.6%)	**< 0.001**
	Squamous cell carcinoma	253	114 (37.3%)	139 (45.4%)	
Histologic grade	G1	19	10 (3.6%)	9 (3.3%)	0.430
	G2	135	63 (23%)	72 (26.3%)	
	G3	119	65 (23.7%)	54 (19.7%)	
	G4	1	0 (0%)	1 (0.4%)	
OS event	Alive	234	125 (40.8%)	109 (35.6%)	**0.043**
	Dead	72	28 (9.2%)	44 (14.4%)	
Radiation therapy	No	122	60 (19.6%)	62 (20.3%)	0.907
	Yes	184	93 (30.4%)	91 (29.7%)	
Primary therapy outcome	PD	23	7 (3.2%)	16 (7.3%)	**0.019**
	SD	6	5 (2.3%)	1 (0.5%)	
	PR	8	2 (0.9%)	6 (2.7%)	
	CR	182	101 (46.1%)	81 (37%)	

**Table 2:** Univariate and multivariate analyses of CESC patient OS.

Characteristics	Univariate analysis			Multivariate analysis		
	Hazard ratio	95% CI	P value	Hazard ratio	95% CI	P value
Age	1.289	0.810-2.050	0.284			
T stage	3.863	2.072-7.201	**<0.001**	5.276	1.251-22.258	**0.024**
N stage	2.844	1.446-5.593	**0.002**	2.017	0.732-5.560	0.175
M stage	3.555	1.187-10.641	**0.023**	0.000	0.000-Inf	0.998
Clinical stage	2.369	1.457-3.854	**<0.001**	0.399	0.077-2.066	0.273
Histological type	1.033	0.543-1.969	0.920			
Histologic grade	0.866	0.514-1.459	0.589			
S100A10	1.293	1.026-1.629	**0.029**	1.263	0.761-2.094	0.366

**Table 3:** The relationship between hypermethylation levels and CESC patient prognostic outcomes.

Name	HR	P.value
5'UTR-N_Shelf-cg04989070	0.715	0.1956
Body-Open_Sea-cg05368119	0.631	0.0531
TSS1500-S_Shore-cg06698332	0.424	**0.0006**
5'UTR-Island-cg06786599	1.887	**0.0072**
TSS200-Island-cg13249591	0.813	0.4087
5'UTR-Island-cg17711091	1.253	0.4381
TSS1500-S_Shore-cg18348690	0.476	**0.0027**
5'UTR-Island-cg18892537	1.434	0.1598
TSS1500-S_Shore-cg20994097	0.593	**0.0389**
TSS200-Island-cg24594295	1.986	**0.0363**
TSS200-Island-cg25848158	1.425	0.2354
5'UTR-N_Shore-cg26230275	0.61	0.1178
